# The Institutes of Medicine

**Published:** 1847-11

**Authors:** 


Art. VIII.—*The Institutes of Medicine.* By Martyn Paine, A.M., M.D., Professor of the Institutes of Medicine and Materia Medica in the University of New York; Member of the Royal Verein fur Heilkunde in Preussen; of the Medical Society of Leipsic; of the Montreal Natural History Society, and other Learned Associations. New York: Harper and Brothers. 8vo. pp. 826. 1847.

Dr. Paine is known as one of the most industrious and voluminous of American writers on medicine. His "*Commentaries,*" in two large volumes, have been for several years before the profession; he is also author of a volume on Materia Medica. In all his works, Dr. Paine exhibits equal industry, learning and singularity. "*Solidism and vitalism,*" as he declares in the first sentence in the volume before us, form the basis of all his medical philosophy. He is no believer in the maxim that truth lies between extremes—*in medio tutissimus ibis*—but is, out and out, a solidist. He will have no terms, no truce with humoralism. "Chemical and mechanical philosophy," in his view, "are strangers to the philosophy of medicine. They have no relationship, no sympathies, but carry on a perpetual hostility."

In this position Dr. Paine stands almost alone, and although he speaks of the system he opposes as tottering to its fall, we do not learn that he is gaining supporters to his standard. On the contrary, from all that we see, the school of which he is so warm and learned an advocate grows continually weaker, insomuch that there is every prospect that the present generation of "vitalists" will be the last.

Dr. Paine is a bold as well as industrious writer, and does not decline the most rigid scrutiny of his doctrines. We admire his spirit, although we cannot always approve the temper exhibited in his discussions. It may be that severity of criticism has provoked him to a harshness of language which he would not have used without provocation, and which certainly is not suited to his subject.

At present we have not room to enter into an examination of his "Institutes," which, we may remark, is a vast repository of facts and arguments, from which the solidist may derive all that can be found to sustain this view of the *philosophy of medicine.* We may return to the volume at another time, and give the reader a fuller view of its singular contents; meantime, all who take an interest in such topics—and we hope the number is large—will apply to the work itself. When we say that it is elaborate and learned, we do the author simple justice, as we believe we do when we add that he is honest and sincere in his convictions, and in all his labors is actuated by love for his profession. Indeed, if he has not used his pen *con amore*, we know not what living writer has. This work is on sale at the bookstores of Messrs. Maxwell and Prescott, Louisville.

